# Assessment of *Borrelia miyamotoi* in febrile patients and ticks in Alsace, an endemic area for Lyme borreliosis in France

**DOI:** 10.1186/s13071-020-04071-9

**Published:** 2020-04-17

**Authors:** Pierre H. Boyer, Joris Koetsveld, Laurence Zilliox, Hein Sprong, Émilie Talagrand-Reboul, Yves Hansmann, Sylvie Josiane de Martino, Nathalie Boulanger, Joppe W. Hovius, Benoît Jaulhac

**Affiliations:** 1 University of Strasbourg, Virulence bactérienne précoce UR7290-Lyme borreliosis group, FMTS - CHRU Strasbourg, Institut de Bactériologie, Strasbourg, France; 2 Center for Experimental and Molecular Medicine, Academic Medical Center, University of Amsterdam, 1105 AZ Amsterdam, The Netherlands; 3 French National Reference Center for Borrelia, Hôpitaux Universitaires de Strasbourg, Strasbourg, France; 4 Centre for Zoonoses & Environmental Microbiology, Centre for Infectious Disease Control, National Institute for Public Health and the Environment, Bilthoven, The Netherlands; 5 Department of Infectious and Tropical Diseases, Hôpitaux Universitaires de Strasbourg, Strasbourg, France

**Keywords:** *Borrelia miyamotoi*, *Borrelia miyamotoi* disease, GlpQ, Tick-borne diseases, Post-tick bite fever

## Abstract

**Background:**

*Borrelia miyamotoi* is a relapsing fever *Borrelia* species transmitted by ticks of the *Ixodes ricinus* complex. Human disease caused by *B. miyamotoi* was first described in Russia and later in the USA and Japan. Additionally, five cases of meningoencephalitis in immunocompromised patients and one case in an apparently immunocompetent patient were described.

**Methods:**

We investigated the presence of *B. miyamotoi* in *I. ricinus* nymphs and in patients suspected of human granulocytic anaplasmosis, in Alsace (France), an endemic area for *I. ricinus* ticks and Lyme borreliosis, using direct (PCR) and indirect diagnosis (glycerophosphoryldiester-phosphodiesterase (GlpQ) serology).

**Results:**

*Borrelia miyamotoi* was found in 2.2% of 4354 ticks collected between 2013 and 2016. None of the 575 blood samples, collected from the patients suspected of HGA, was found positive for *B. miyamotoi* by PCR. Acute and late sera from 138 of these 575 patients were available. These paired sera were tested for IgM and IgG antibodies against the *B. miyamotoi* GlpQ antigen. A total of 14 out of 138 patients had at least one positive parameter (i.e. anti-GlpQ IgG and/or IgM). One patient seroconverted for IgG, and three had isolated IgM in the acute serum. These three patients were treated with doxycycline which could have prevented seroconversion. After reviewing clinical data and other biological tests performed, co-exposure among different microorganisms vectored by ticks or serological cross-reactivity could not be ruled out in these different cases. One patient had persistent IgG, which strongly suggests previous exposure to *B. miyamotoi*.

**Conclusions:**

Humans can be exposed to *B. miyamotoi* through tick bites in Alsace. We present serological data for possible *B. miyamotoi* exposure or infection of patients with fever after tick bite. Future studies should determine the incidence, clinical course and burden of this emerging tick-borne disease in other parts of Western Europe.
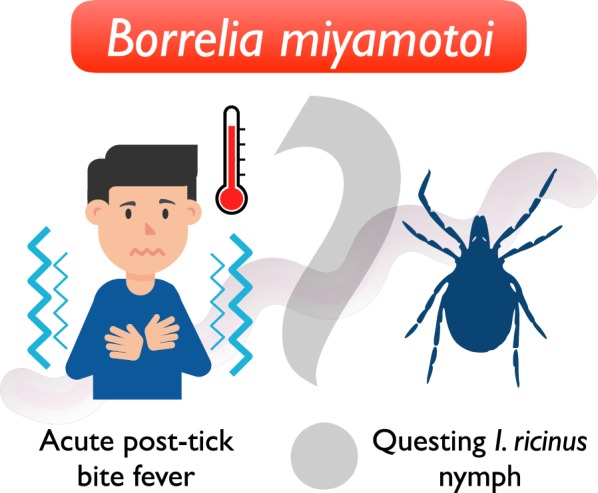

## Background

*Borrelia miyamotoi* is currently the only *Borrelia* species belonging to the relapsing fever group that is transmitted by ticks of the *Ixodes ricinus* complex [1]. In 2011, the first series of patients with febrile diseases caused by *B. miyamotoi* were described in Russia [2] and later in the USA [3–5]. The disease was designated as *Borrelia miyamotoi* disease (BMD) or hard tick-borne relapsing fever and should be the object of differential diagnosis of human granulocytic anaplasmosis (HGA) [3]. In parallel, cases of meningoencephalitis caused by *B. miyamotoi* in highly immunocompromised patients, receiving B-cell depleting therapy have been described since 2013 and one case was reported in an apparently immunocompetent patient [6–9]. In central Europe, only one blood sample has been found to be PCR-positive so far, albeit in a person without symptoms [10]. However, serological evidence for exposure was found among forestry workers [11]. More recently, a case of post-tick bite febrile syndrome has been reported in western Europe, and serological results suggested that *B.* *miyamotoi* was the causative agent of the patient’s symptoms [12].

The Alsace region of France is an area with a high density of *I. ricinus* [13–15]. Since *B. miyamotoi* was found in *I. ricinus* ticks in France and surrounding countries [16–18], we aimed to study the prevalence of *B. miyamotoi* in patients suspected of post-tick bite febrile illness in northeastern France using direct and indirect diagnostic tools, as well as by measuring *B. miyamotoi* infection rates in *I. ricinus* ticks collected in the same region.

## Methods

### Study area and tick collection

Alsace is a region located in the northeastern part of France, bordering Germany. Four collection sites were investigated in different locations in the region, with variable vegetation and environment (i.e. natural or suburban). These sites were defined in previous studies [13, 19], and details are shown in Additional file 1: Table S1. Among these four sites, site A was defined as the control site because of the low prevalence of Lyme borreliosis in this area [13, 19].

From April 2013 to November 2016, 4354 questing *I. ricinus* nymphs were field collected by dragging a white flannel flag (1 × 1 m) over low vegetation.

### Patients and whole blood samples

Between May 2010 and July 2016, EDTA blood samples from 575 patients were sent to the clinical microbiology laboratory of the University Hospitals of Strasbourg for *Anaplasma phagocytophilum* diagnosis by PCR. From May 2010 to July 2012, these patients were recruited for the study of HGA [20]. The inclusion criteria were (i) fever or another symptom presumed to be related to a tick bite occurring within a maximum of 4 weeks before the beginning of symptoms; (ii) patients exposed to tick bites with fever and at least one haematological abnormality or abnormal liver function tests; and (iii) patients with at least one haematological abnormality or abnormal liver function tests occurring maximum 4 weeks after a proven tick bite. At the time of recruitment, patients were examined by infectious disease specialists, and an aetiological investigation was thoroughly conducted to establish a differential diagnosis. Clinical data at the time of examination were obtained by a standardized questionnaire.

For the period from July 2012 to July 2016, all the blood samples sent to Strasbourg University laboratory for investigation of *A. phagocytophilum* by PCR were examined. They were issued from the same hospitals as in the previous period, but clinical data were not obtained.

### Serum samples

For a subset of 138 of the 575 patients presented above, both acute and convalescence sera were available. Serum samples were obtained at the same time as the EDTA whole blood samples for PCR. Additionally, 50 healthy blood donors from Alsace and 50 donors from a non-endemic region for Lyme borreliosis, were used as negative controls to determine the serological background in the French population.

Blood samples stored at − 80 °C and serum samples stored at − 30 °C were retrospectively analysed by real-time PCR and serology respectively.

### Pan-relapsing fever *Borrelia* PCR

Detection of relapsing fever (RF) *Borrelia* was performed by real-time PCR adapted from a protocol published by Hovius et al. [6] targeting a 200-bp fragment of the *16S* rDNA. Each positive sample was sequenced by the GATC Biotech company (Mulhouse, France) using the Sanger method with the primers F-relfev and R-relfev.

Bacterial identification was performed using bacterial phylogenic analysis with the Bioinformatics Bacterial Identification tool (leBIBI^QBPP^) [21].

As a confirmation assay, a second *B. miyamotoi* real-time PCR test, targeting a 116-bp fragment of the flagellin gene of *B. miyamotoi*, was performed on a subset of 135 nymphs [6].

### *Borrelia miyamotoi* GlpQ serology

Serum reactivity against recombinant GlpQ was tested for IgM and IgG antibodies with an ELISA technique as previously described [22, 23]. The cut-off values were calculated as the mean result plus three times the standard deviation of the 10 negative controls included in each experiment. All the sera were tested twice in separate experiments. Positive sera were subsequently tested by western blot as previously described [22]. IgG and IgM serology was considered as positive when both ELISA and western blot were deemed positive.

### BMD case definition

BMD cases were divided upfront into three categories according to the level of evidence of the technique used (i.e. molecular biology or serology). Cases proven by both PCR methods were defined as confirmed cases. Serological results were interpreted according to the delay between the onset of the symptoms and the sampling of the serum. Contact with *B. miyamotoi* was considered as probable in patients who seroconverted whatever the result of the anti-GlpQ IgM in the acute serum. Exposure to *B. miyamotoi* was considered as possible in patients with solitary IgM in sera obtained within 11–20 days after the onset of the symptoms (a compatible delay for the only appearance of anti-GlpQ IgM antibodies) [22] and treated with doxycycline, which could abort seroconversion [24]. Solitary IgM or persistent IgM positivity was classified as false IgM results, when there was evidence of an infection with other microorganisms (e.g. cytomegalovirus or Epstein-Barr virus) which are known to give a polyclonal response in serological assays. Finally, patients with persistent IgG were considered as previously exposed to *B. miyamotoi*.

### Statistical analysis

Ticks infection rates were calculated and expressed as percentages with their 95% confidence interval. Statistical modelling using a generalized linear model was performed to assess *B. miyamotoi* prevalence in ticks in these four different locations. Data were analysed using R Studio R version 3.4.0 (2017-04-21, https://cran.r-project.org/).

## Results and discussion

### *Borrelia miyamotoi* vector epidemiology in Alsace

All the 4354 nymphs collected during the years of field sampling were morphologically identified as *I. ricinus* nymphs. Among these 4354 nymphs, 2.18% (95% CI: 1.77–2.67) (n=94) were positive by pan-RF *Borrelia* PCR. Amplicon sequences obtained by the *16S* rDNA PCR assay allowed the identification of *B. miyamotoi*, and no other RF *Borrelia* species was identified. The *B. miyamotoi*-specific confirmatory PCR assay performed on a subset of 135 nymphs was 100% consistent with the identification obtained by the *16S* rDNA PCR. Thirteen nymphs were positive and 122 were negative according to both techniques, which indicates neither a lack of sensitivity nor lack of specificity.

The generalized linear model revealed a disparity in *B. miyamotoi* prevalence in *I. ricinus* ticks among the four collection sites (Additional file 1: Table S1). Indeed, the odds ratio of *B. miyamotoi* infection rate was significantly higher in site B (OR: 2.718, 95% CI: 1.218–6.069) than for the control site A. The detailed prevalence rates over four years and on the four collection sites are shown in Additional file 2: Table S2. No significant difference in prevalence was observed among the four years of collection regardless of the considered site.

The prevalence (2.18%) of *B. miyamotoi* found in Alsace among *I. ricinus* nymphs is consistent with the findings of other studies conducted in regions near Alsace [16, 17] and elsewhere in the Northern hemisphere [2, 25–27], as well as in regions where BMD is regularly described [2]. Collection site B showed a higher *B. miyamotoi* infection rate in *I. ricinus* nymphs; since *B. burgdorferi* (*s.l*.) was also found to have a higher prevalence at this collection site [13, 19], this result is in line with previous studies showing a correlation between infection rates of *B. miyamotoi* and *B. burgdorferi* (*s.l*.) in *Ixodes* ticks [28].

Interestingly, the prevalence of *B. miyamotoi* (2.18%) among ticks in Alsace is close to the prevalence rates of *A. phagocytophilum* in this area (ranging from 0.4% to 1.2%) [13]. This latter microorganism causes a similar febrile syndrome and is mainly diagnosed using molecular tools [20].

### *Borrelia miyamotoi* in febrile patients after a tick bite

#### Patients

Whole blood samples of 575 patients were sent to our laboratory for the detection of *A. phagocytophilum* by PCR; clinical data were available for 155 patients. Among these 155 patients, 89 remembered a tick bite, 131 had fever at the time of sampling, and the other patients had at least one other symptom occurring after a tick bite: articular pain, meningeal signs and headache. One hundred and one patients had hepatic or haematological abnormalities on the routine laboratory tests.

#### Whole blood PCR

None of the 575 whole blood samples were found to be positive by pan-RF *Borrelia* PCR. Consequently, no confirmed clinical cases of BMD could be evidenced by PCR methods in our cohort. This fact is corroborated by large-scale studies on post-tick bite febrile illnesses. Indeed, a very small proportion of the tested blood samples were found to be PCR-positive for *B. miyamotoi* in these previous studies with rates of 0.16% (1/626), 0.84% (97/11,515), 0.33% (7/2150), 0.11% (8/7292) and 0.49% (2/408) [3, 4, 10, 29, 30]. In contrast, in Russia, 15.2% (46/302) of the post tick-bite febrile patients [2] were found PCR-positive for *B. miyamotoi*. In the Russian study [2] blood samples were all collected during febrile episodes which is consistent with recent observations demonstrating that *B. miyamotoi* spirochetemia only occurs during febrile episodes [31]. Moreover, Molloy et al. [3] have shown that spirochetemia is lower during *B. miyamotoi* infection (7787 spirochetes/ml) than during other RF *Borrelia* which can reach up to 100,000 spirochetes/ml [32]. Consequently, sampling during febrile episodes is important since it maximizes the likelihood of having PCR positive proven infection. Because not all acute samples were drawn during febrile episodes (median time of sampling after the onset of fever: 6 days (IQR: 4–12)), this is a limitation of the present study.

#### Serological study

All serum samples were tested to assess their IgM and IgG reactivity against *B. miyamotoi* GlpQ with ELISAs. The median time of sampling after the onset of fever was 6 days (IQR: 4–12) for acute samples. Convalescence samples were collected at a median of 6.28 weeks (IQR: 5.96–7.04) after the acute serum.

In total, 14/138 patients had at least one positive parameter (i.e. anti-GlpQ IgG and/or IgM) using the two-tiered algorithm (Table 1).

**Table 1:** GlpQ serology kinetics for the 14/138 patients with a serological follow-up, who had at least one positive (ELISA + WB) test and their doxycycline treatment status after the acute blood sampling

Patient	Age (years)	Time between symptom onset and acute sampling (day)	Time between symptom onset and convalescence sampling (days)	Time between the two samples (days)	Tick bite	Serological results				Doxycycline administration after the acute sampling	Final diagnosis retained by physician
						Acute sample IgM	Acute sample IgG	Convalescence sample IgM	Convalescence sample IgG		
A	65	17	63	46	Yes	Neg	Neg	**Pos**	**Pos**	Yes	**HGA**
B	37	8	42	34	No	**Pos**	Neg	Neg	Neg	Yes	**Malaria**
C	67	14	56	42	Yes	**Pos**	Neg	Neg	Neg	Yes	**HGA**
D	83	9	42	33	Yes	**Pos**	Neg	Neg	Neg	Yes	**HGA**
E	44	4	56	52	Yes	Neg	Neg	**Pos**	Neg	Yes	**No**
F	62	Unknown	Unknown	82	Unknown	Neg	**Pos**	Neg	**Pos**	Unknown	Unknown
G	49	16	56	40	Yes	Neg	Neg	**Pos**	Neg	Yes	**No**
H	53	Unknown	Unknown	50	Unknown	Neg	Neg	**Pos**	Neg	Unknown	Unknown
I	38	Unknown	Unknown	50	Unknown	Neg	Neg	**Pos**	Neg	Unknown	Unknown
J	42	Unknown	Unknown	40	Unknown	**Pos**	Neg	Neg	Neg	Unknown	Unknown
K	71	18	79	61	No	**Pos**	Neg	**Pos**	Neg	Yes	**CMV infection**
L	53	19	56	37	No	**Pos**	Neg	Neg	Neg	No	**EBV infection**
M	28	17	70	53	No	**Pos**	Neg	Neg	Neg	No	**CMV+EBV infection**
N	39	5	49	44	Yes	Neg	Neg	**Pos**	Neg	No	**No**

*Abbreviations*: HGA, human granulocytic anaplasmosis; EBV, Epstein-Barr virus; CMV, cytomegalovirus; Pos, positive; Neg, negative; No, no final diagnosis retained at time of consultation

Patient A seroconverted between the acute and the late serum sampling in IgG and IgM. According to our definitions, exposure to *B. miyamotoi* can be considered as probable. However, this patient was found to be PCR-positive for *A. phagocytophilum* in the acute blood sample. Several hypotheses can explain these results. First, co-infection by *A. phagocytophilum* and *B. miyamotoi* could have occurred; although these are two rare tick-borne microorganisms, some *I. ricinus* ticks were found to be positive for these two microorganisms [33]. Moreover, the acute serum was sampled 17 days after the disease onset and did not show a trace of anti-GlpQ IgM. Successive exposure to *A. phagocytophilum* and *B. miyamotoi* is more likely to explain these results, especially as convalescence serum for *B. miyamotoi* was sampled nine weeks after the disease onset and because this person very often visited the forest. This seroconversion cannot be directly linked to a disease.

Patient B was found to have malaria because *Plasmodium falciparum* trophozoites were found in his blood smear, and a specific PCR assay for *P. falciparum* was positive in the acute sample. He was a soldier returning from a mission in an African country; since his return, he had regularly run in a forest in Alsace. Cross-reactivity leading to false positivity could explain these serological results. However, it should be mentioned that he could have had a possible contact with a RF *Borrelia* in Africa or with *B. miyamotoi* which could have given a positive anti-GlpQ IgM result in the acute serum without IgG seroconversion since he had been treated with doxycycline.

Patients C and D were found to have HGA; indeed, *A. phagocytophilum* PCR was positive in the acute sample. Very interestingly, they received doxycycline after the sampling of their acute sera. Doxycycline can prevent seroconversion in Lyme borreliosis [24] and could also have prevented the appearance of anti-GlpQ IgG in these patients. Here, again, serology evidenced a possible exposure to *B. miyamotoi*, but cross-reactivity leading to false positivity cannot be ruled out in these three patients. For patient J, the presence of isolated IgM without IgG seroconversion must be interpreted with caution; no clinical data could be collected concerning this case, which makes interpretation difficult.

Among the other ten patients out of the subset of 138, cross-reactivity is highly probable for patients L and M who had isolated IgM in their acute sera similar to patient K who had persistent anti-GlpQ IgM. Indeed, patient L had a serological profile compatible with a primary EBV infection. Patient M had a positive PCR result for both EBV and CMV in the whole blood and CMV-positive PCR bronchoalveolar lavage fluid, and his clinical state was improved with foscarnet. The antibody response to these two viruses (EBV and CMV) is known to give false-positive results in serology, and these two agents more likely to explain the patient’s symptoms. For patient K, the diagnosis of a CMV infection was retained.

For patients G, H and I who had isolated IgM in their convalescence sera, the results are difficult to interpret and can hardly be linked to the acute event of fever since the convalescence sera were sampled about two months after the acute sera. Indeed, anti-GlpQ IgM reaches a peak approximately 11 and 20 days after disease onset, and anti-GlpQ IgG peaks between 21 and 50 days after the onset [22]. Cross-reactivity can explain these symptoms, but possible exposure to *B. miyamotoi* without disease could also be considered as the patients’ clinical state was good during the sampling of the late sera.

Interestingly, the acute serum of patients E and N was taken only four and five days respectively, after the disease onset. Within four days, the IgM response might not have fully developed, and thus could have only been detected during the convalescence blood sampling.

Finally, persistent IgG were observed in patient F indicating that he was probably previously exposed to *B. miyamotoi*.

The serological evidence provided by our study does not allow us to establish with certainty that the febrile syndromes are linked to *B. miyamotoi* infection as PCR would allow. However, collectively these results suggested possible exposure to *B. miyamotoi* in this cohort of patients as it has already been reported in the Netherlands [11], indicating that BMD could have been involved in the reported febrile episodes in some of these patients.

## Conclusions

In this study we analyzed samples from 575 post-tick bite febrile patients and 4354 *I. ricinus* nymphs. We were able to detect *B. miyamotoi* in ticks collected in the Alsace region (France) with an infection rate (2.18%) consistent with tick infection rates in endemic regions of BMD in Russia and other areas in Europe. We were unable to detect *B. miyamotoi* by PCR in a cohort of 575 patients with fever after a tick bite. However, GlpQ serology suggested a possible *B. miyamotoi* exposure or acute infection in 14 of 138 patients.

## Supplementary information

**Additional file 1: Table S1.** Multivariate analysis of *B. miyamotoi* prevalence among the four collection sites in Alsace and their GPS coordinates. Results are expressed using the odds ratio and its 95% confidence interval (95% CI).

**Additional file 2: Table S2.** Proportion and number (*n*) of *B. miyamotoi* infected nymphs and its 95% confidence interval (95% CI), among the collected nymphs (N) at the four sites during the four years of collection.

## Data Availability

All relevant data are included in the manuscript. Ten *B. miyamotoi 16S* rDNA sequences were deposited in the GenBank database under the accession numbers MN695028-MN695037.

## Abbreviations

BMD: *Borrelia miyamotoi* disease
CMV: Cytomegalovirus
DNA: deoxyribonucleic acid
EBV: Epstein-Barr virus
EDTA: ethylenediaminetetraacetic acid
GlpQ: glycerophosphodiester phosphodiesterase
HGA: human granulocytic anaplasmosis
PCR: polymerase chain reaction
rDNA: ribosomal DNA
RF: relapsing fever
