# Supplementary material for: Assessment of Borrelia miyamotoi in febrile patients and ticks in Alsace, an endemic area for Lyme borreliosis in France
Source: Parasit Vectors. 2020 Apr 17;13:199. doi: 10.1186/s13071-020-04071-9 (PMC7165395; doi:10.1186/s13071-020-04071-9)
Supplement: Supplementary file 1 — Additional file 1: Table S1. Multivariate analysis of B. miyamotoi prevalence among the four collection sites in Alsace and their GPS coordinates. Results are expressed using the odds ratio and its 95% confidence interval (95% CI). [file 13071_2020_4071_MOESM1_ESM.docx]

| **Site** | **GPS coordinates** | **odds ratio** | **CI_95%_** | **p-value** |
| --- | --- | --- | --- | --- |
| A | 47°42’03.0"N  7°08’31.0"E | reference | reference | reference |
| B | 47°55’08.3"N  7°12’37.6"E | 2.718 | 1.218 - 6.069 | 0.015 |
| C | 48°26’9.503’’N  7°24’42.181”E | 1.375 | 0.571 - 3.309 | 0.478 |
| D | 48°31’07.1"N  7°44’36.0"E | 2.088 | 0.898 - 4.856 | 0.087 |

Additional file 1 : Table S1. Multivariate analysis of *B. miyamotoi* prevalence among the four collection sites in Alsace and their GPS coordinates. Results are expressed using the *odds ratio* and its 95% confidence interval (CI 95%).
