# Supplementary material for: Assessment of Borrelia miyamotoi in febrile patients and ticks in Alsace, an endemic area for Lyme borreliosis in France
Source: Parasit Vectors. 2020 Apr 17;13:199. doi: 10.1186/s13071-020-04071-9 (PMC7165395; doi:10.1186/s13071-020-04071-9)
Supplement: Supplementary file 2 — Additional file 2: Table S2. Proportion and number (n) of B. miyamotoi infected nymphs and its 95% confidence interval (95% CI), among the collected nymphs (N) at the four sites during the four years of collection. [file 13071_2020_4071_MOESM2_ESM.docx]

| **Site** | | **2013** | | | **2014** | | | **2015** | | | | **2016** | | | | **All years** | | | |
| --- | --- | --- | --- | --- | --- | --- | --- | --- | --- | --- | --- | --- | --- | --- | --- | --- | --- | --- | --- |
|  |  | N | Proportion % (n) | CI _95_ % | N | Proportion % (n) | CI _95_% | N | Proportion % (n) | CI _95_% | N | | Proportion % (n) | CI _95_% | N | | Proportion % (n) | CI _95_% |  |
| A | 69 | 1.45 (1) | [0.04; 7.81] | 134 | 0.75 (1) | [0.02; 4.09] | 192 | 1.04 (2) | [0.13; 3.71] | 230 | | 1.3 (3) | [0.27; 3.76] | 625 | | 1.12 (7) | [0.45; 2.24] |  |  |
| B | 400 | 2.75 (11) | [1.38; 4.87] | 417 | 2.16 (9) | [0.99; 4.06] | 299 | 3.01 (9) | [1.38; 5.64] | 357 | | 4.2 (15) | [2.37; 6.83] | 1,473 | | 2.99 (44) | [2.18; 3.99] |  |  |
| C | 374 | 1.6 (6) | [0.59 ; 3.46] | 219 | 1.83 (4) | [0.50 ; 4.61] | 189 | 3.17 (6) | [1.17 ; 6.78] | 392 | | 0.6 (2) | [0.06 ; 1.83] | 1,174 | | 1.53 (18) | [0.91 ; 2.41] |  |  |
| D | 256 | 1.56 (4) | [0.43 ; 3.95] | 297 | 2.02 (6) | [0.74 ; 4.34] | 276 | 2.9 (8) | [1.26 ; 5.63] | 253 | | 2.79 (7) | [1.12 ; 5.62] | 1,082 | | 2.31 (25) | [1.50 ; 3.40] |  |  |
| All sites | 1,099 | 2.00 (22) | [1.26; 3.01] | 1,067 | 1.87 (20) | [1.15; 2.88] | 956 | 2.62 (25) | [1.70; 3.84] | 1,232 | | 2.29 (27) | [1.45; 3.17] | 4,354 | | 2.18 (94) | [1.77; 2.67] |  |  |

Additional file 2 : Table S2. Proportion and number (n) of *B. miyamotoi* infected nymphs and its 95% confidence interval (CI 95%), among the collected nymphs (N) at the four sites during the four years of collection.
